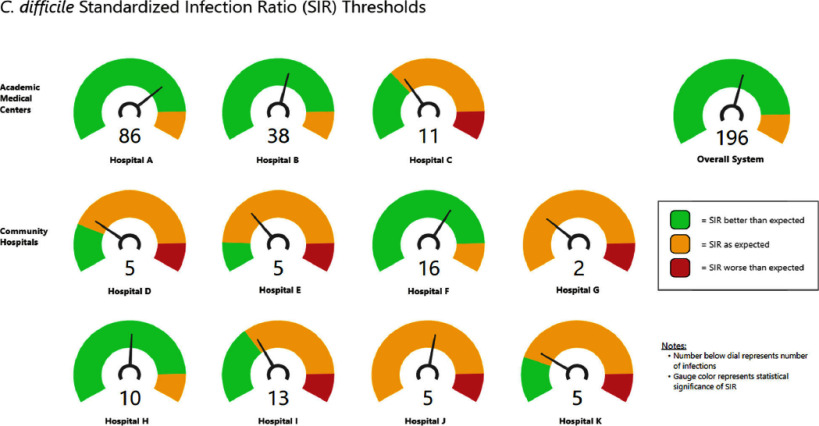# Defining Thresholds to Identify Hospitals in a Healthcare System with Opportunities to Significantly Improve C. difficile Infections

**DOI:** 10.1017/ash.2025.211

**Published:** 2025-09-24

**Authors:** Peter Batten, Aleah King, Patrick Gordon, Sharon Wright

**Affiliations:** 1 Beth Israel Lahey Health

## Abstract

**Background:** Clostridioides difficile infections (CDI) are associated with patient morbidity and mortality and also may impact reputational and financial metrics. CDI was identified as a healthcare-associated infection of concern at several of the hospitals in our healthcare system and set as a target for improvement. We sought to create an easily interpretable tool to help select hospitals with the greatest opportunity to benefit from this work. **Methods:** The National Healthcare Safety Network’s (NHSN) data (infection counts and number of predicted infections) for LabID CDI from Oct 2023-Sept 2024 were exported for 3 academic and 8 community hospitals in our healthcare system in eastern Massachusetts and New Hampshire. Using published source code from the Centers for Disease Control and Prevention recreated in R software (v.4.3.3), we calculated the statistical significance of the Standardized Infection Ratio (SIR) relative to 1.0 using the p-value. We then performed the statistical test iteratively by adjusting the number of infections by one in each direction from the true observed number of infections, to establish thresholds for significantly improved or worsened performance. Data were displayed as gauge charts with indication of current SIR statistical significance defined by color (red, yellow and green) and distance to threshold that would alter that significance (See Figure). Viable opportunities for improvement were defined as being within 5 or fewer infections of calculated thresholds. **Results:** Review of CDI data across all 11 sites demonstrated more improvement opportunities at some sites than others. Four sites were in green ranges, seven in yellow and none in red. All opportunities for viable improvement were identified in the yellow range; 5 of 11 sites had potential for improvement in SIR (Hospitals C, D, E, I, K) and 2 for worsening of SIR (Hospitals G, J); see Figure. **Conclusion:** In our healthcare system, this model provided insight into site-specific opportunities for improvement in CDI by highlighting sites closest to achieving a statistically significant change in SIR. Although factors such as morbidity and cost may influence selection of targets for improvement, visual depiction of viable thresholds for change in SIR may provide an indicator of facilities likely to yield the most benefit relative to investment required for reduction efforts.

**Figure:**